# Optimization of the *in vitro* oxidative biotransformation of glimepiride as a model substrate for cytochrome p450 using factorial design

**DOI:** 10.1186/2008-2231-20-38

**Published:** 2012-09-10

**Authors:** Dipti B Ruikar, Sadhana J Rajput

**Affiliations:** 1 Quality Assurance Laboratory, Centre Of Relevance And Excellence In Novel Drug Delivery System, Pharmacy Department, G. H. Patel Pharmacy Building, Donor’s Plaza, The Maharaja Sayajirao University Of Baroda, Fatehgunj, Vadodara, 390 002, Gujarat, India

**Keywords:** Incubation time, Human liver microsomes, Substrate, Turnover rate, Contour plot

## Abstract

**Background and purpose of the study:**

Glimepiride (GLM) was chosen as a model substrate in order to determine the kinetic parameters for *in vitro* metabolism via human liver micrososmes (HLM). We aimed to optimize the turnover of the substrate by the test system in relation to incubation time and HLM concentration in such a way that it was linearly dependent on time and less than 20% of the substrate was consumed which utilized the lowest amount of the HLM. Further we aimed to report K_m_ and V_max_ values for GLM.

**Methods:**

Linearity of enzyme reactions in microsomal incubations was assessed by monitoring the effect of incubation time (from 5 to 60 min) and HLM concentration (from 0.2 to 0.75 mg/ml) on metabolite formation of GLM. The ideal conditions for turnover of GLM were justified using 3x3 factorial design. F value was calculated to confirm the omission of insignificant terms from the full-model to derive a reduced- model polynomial equation. The regression equation was used to develop a contour plot that showed turnover rate within the limits of this design. The optimized reaction velocity data was extrapolated to carry out the kinetic studies *in vitro* to generate a saturation curve for the determination of K_m_ and V_max_ values.

**Results:**

The reaction was found to be linear with respect to both incubation time between 24 and 50 min and HLM concentration between 0.3 to 0.65 mg/ml. The K_m_ and V_max_ values obtained by nonlinear least squares regression method was found to be 28.9 ± 2.97 μMole and 0.559 ± 0.017 μMole respectively. Lineweaver-Burk plot was also used to estimate K_m_ and V_max_ which yield value of 29.411 ± 1.25 μMole and 0.571 ± 0.020 μMole/min/mg protein respectively.

**Major conclusion:**

The statistical approach successfully allows for the optimization of reaction time course experiments. The results obtained with linear as well as the nonlinear transformation were found to be in close agreement with each other which shows the best precision for estimates of K_m_ and V_max_.

## Background

For most drugs, biotransformation is the major route of elimination, and oxidative metabolism by cytochrome P450 (CYP450) enzymes is a common metabolic pathway [1]. More than 90% of oxidative metabolic reactions (phase I) of drugs are catalyzed by enzymes of the CYP450 family present in liver [2]. In order to investigate drug metabolism prior to human exposure, there are a number of options ranging from *in vitro* screening with human enzymes to *in vivo* assessment in experimental animals. Although animal models can provide information about the biochemical potential for drug biotransformation (i.e. identifying the metabolite(s) that can be formed), such models may only indicate what is biologically possible, not what is biologically relevant for human drug exposure? This is due to the well documented inter-species differences in both expression and substrate specificity of drug metabolizing enzyme. Thus human tissue systems called as human liver microsomes have been developed to address the limitations of animal models of drug metabolism [3,4]. The Pharmaceutical Research and Manufacturers of America Perspective (PhRMA) and U.S. Food and Drug Administration (USFDA) guidelines address the specific designs of the studies, to define a minimal best practice for *in vitro* and *in vivo* pharmacokinetic studies targeted to the development [5,6].

Determinations of the initial velocity conditions are important for the accurate investigation of enzyme kinetic parameters. To determine the kinetic parameters for CYP450 substrate metabolism, the turnover of the substrate by the test system needs to be optimized such that it is linearly dependent on time and less than 20% of the substrate is consumed. Also it is desirable to utilize the lowest amount of enzyme in the incubation that yields readily quantifiable metabolite concentrations. A concentration of below 0.5 mg/ml microsomal protein is suggested as the low enzyme concentration would help maintain minimal enzyme binding. The initial rates of drug disappearance in *in vitro* metabolism are to be measured for minimizing any discrepancy caused by the difference in drug concentrations at the start and at the time of measurement [7]. Once the initial velocity conditions have been established, the substrate concentration should be varied to generate a saturation curve for the determination of K_m_ (substrate affinity of the enzyme) and V_max_ (maximum reaction rate) values.

To evolve an ideal incubation condition it is important to understand the complexity of enzymatic reactions in a more systematic way using established statistical tools such as full factorial design. Thus the usual approach was to start with a screening design including all controllable factors that may possibly influence the experiment, identify the most important ones and proceed with a 3X3 experimental optimization design [8,9].

GLM belongs to second generation sulphonylurea which is being used for the treatment of non-insulin dependent diabetes mellitus (NIDDM), to achieve appropriate control of blood glucose level. In addition, it maintains a better physiological regulation of insulin secretion than other sulphonylurea during physical exercise. GLM has been shown to undergo hepatic oxidative biotransformation via CYP450 system and its metabolism also has been reported using CYP specific species of seven CYP2C9 variants found in Japanese subjects [10-12].

Liquid chromatography with ultraviolet (LC/UV) [13,14], fluorescence [15,16] or mass spectrometry (MS) detection [17,18] has been commonly used for quantitative determination of CYP probe substrates. LC/MS has the advantages of high sensitivity, selectivity and speed. However, LC/MS instrumentation is costly and may not be available for routine analysis in every research laboratory. In addition, the LC/MS-based assays often require the use of different ionization and ion detection modes due to the diverse structure of CYP probe substrate, which creates difficulty and complexity in developing LC/MS methods for simultaneous analysis. Fluorescence and UV are conventional and inexpensive detectors for LC. Fluorescence detectors are very sensitive but respond only to the few analytes that fluoresce. In contrast, many compounds can absorb ultraviolet light. Therefore, LC with UV detection can be used for the analysis of CYP probe substrates and metabolites [19]. The drawback of UV detection is its relatively low sensitivity and selectivity. However, our preliminary results show that the sensitivity of LC/UV is sufficient for the detection of GLM oxidative biotransformation resulting from normal microsomal incubations.

The present investigation justifies using GLM as a model substrate to statistically optimize its oxidative biotransformation *in vitro* within the limits of developed assay design.

## Methods

### Chemicals and reagents

GLM was received as a gift sample from Cadila Healthcare Ltd., Ahmedabad, India. Nicotinamide Adenine Dinucleotide Phosphate, reduced tetra sodium salt (NADPH), Magnesium chloride (MgCl_2_) was purchased from Himedia laboratories, India. Ethylene diamine tetra acetic acid (EDTA), dipotassium hydrogen phosphate and potassium dihydrogenphosphate were purchased from S.d Fine-Chem Limited, India. Methanol and Acetonitrile of HPLC grade were purchased from Spectrochem India. All other chemicals and reagents used in this study were of analytical grade.

### Microsomal source

A pool of the 50 HLM (0.5 ml at 20 mg/ml), mixed gender, in a suspension medium of 250 mM sucrose was obtained from Xenotech LLC., USA and stored at −80°C in a deep freezer. The frozen microsomes were thawed by placing the vial under cold running water and kept in an ice water bath until use. The total CYP450 content, protein concentrations, and specific activity of each CYP450 isoforms were as supplied by the manufacturer.

### Factorial design and optimization

Based on the results obtained in the preliminary experiments, drug concentration, HLM concentration and incubation time were found to be major variables affecting metabolism of GLM. Hence 3X3 factorial design was applied to find the optimized condition for carrying out a reaction time course experiment for GLM’s oxidative biotransformation. In all the experiments NADPH concentration was 1 mM and buffer concentration was 50 mM. In this experimental design, GLM in the presence of HLM was incubated in 27 different combinations.

### Effect of variables

To study the effect of variables, different batches were prepared by using 3X3 factorial design. Drug concentration (X1), incubation time (X2) and HLM concentration (X3) were selected as three independent variables. The independent variable and their levels are shown in Table 1. The turnover rate (Y1%) was taken as a response parameter as the dependent variable. These three factors were evaluated each at 3 levels and experimental trials were performed for all 27 possible combinations as reflected from Table 2. The values of the factors were transformed to allow easy calculation of co-efficient in polynomial equation. Interactive multiple regression analysis and F statistics were utilized in order to evaluate the response. The regression equation for the response was calculated using the following equation-Response: $\text{Y}1\text{(\%)}=\beta_{0}+\beta_{1}\text{X}1+\beta_{2}\text{X}2+\beta_{3}\text{X}3+\beta_{4}\text{X}1^{2}+\beta_{5}\text{X}2^{2}+\beta_{6}\text{X}3^{2}+\beta_{8}\text{X1X}2+\beta_{9}\text{X1X}3+\beta 10\text{X2X}3+\beta_{11}\text{X1X2X}3$ where Y1 (%) is turnover rate and indicates the quantitative effect of the independent variables X1, X2 and X3, which represent the drug concentration, incubation time and HLM concentration respectively, β0 is the intercept while β1- β11 represents the regression coefficient of the system. To identify the significant terms, the variables having p value > 0.05 in the full model were discarded and then the reduced model was generated for the independent variables [20,21].

**Table 1:** Factors, their levels, and coded values

	**Levels**		
**Variables**	**Low**	**Medium**	**High**
Drug concentration (X1)	10 μmole	20 μmole	30 μmole
Incubation time (X2)	10 min	35 min	60 min
HLM concentration (X3)	0.25 mg/ml	0.5 mg/ml	0.75 mg/ml
Coded values	−1	0	+1

**Table 2:** Different batches with their experimental coded level of variables for full factorial design

**Batch no.**	**X1**	**X2**	**X3**	**X**_**1**_^**2**^	**X**_**2**_^**2**^	**X**_**3**_^**2**^	**X**_**1**_**X**_**2**_	**X**_**1**_**X**_**3**_	**X**_**2**_**X**_**3**_	**X**_**1**_**X**_**2**_**X**_**3**_	**% Turnover rate ± (SEM)†**
1	−1	−1	−1	1	1	1	1	1	1	−1	5.01(0.44)
2	−1	−1	0	1	1	0	1	0	0	0	6.5(0.21)
3	−1	−1	1	1	1	1	1	−1	−1	1	8.9(0.41)
4	−1	0	−1	1	0	1	0	1	0	0	8.92(0.25)
5	−1	0	0	1	0	0	0	0	0	0	19.91(0.69)
6	−1	0	1	1	0	1	0	−1	0	0	18.01(0.48)
7	−1	1	−1	1	1	1	−1	1	−1	1	33.4(0.76)
8	−1	1	0	1	1	0	−1	0	0	0	37.69(0.91)
9	−1	1	1	1	1	1	−1	−1	1	−1	38.81(0.56)
10	0	−1	−1	0	1	1	0	0	1	0	4.4(0.84)
11	0	−1	0	0	1	0	0	0	0	0	6.1(0.58)
12	0	−1	1	0	1	1	0	0	−1	0	8.45(0.76)
13	0	0	−1	0	0	1	0	0	0	0	8.05(0.51)
14	0	0	0	0	0	0	0	0	0	0	18.91(0.62)
15	0	0	1	0	0	1	0	0	0	0	15.05(0.65)
16	0	1	−1	0	1	1	0	0	−1	0	31.75(0.53)
17	0	1	0	0	1	0	0	0	0	0	38.45(1.03)
18	0	1	1	0	1	1	0	0	1	0	38.15(0.89)
19	1	−1	−1	1	1	1	−1	−1	1	1	3.8(0.75)
20	1	−1	0	1	1	0	−1	0	0	0	5.24(0.92)
21	1	−1	1	1	1	1	−1	1	−1	−1	7.91(0.72)
22	1	0	−1	1	0	1	0	0	0	0	7.56(0.55)
23	1	0	0	1	0	0	0	0	0	0	19.08(0.78)
24	1	0	1	1	0	1	0	0	0	0	14.32(0.43)
25	1	1	−1	1	1	1	1	−1	−1	−1	30.56(0.67)
26	1	1	0	1	1	0	1	0	0	0	35.91(0.48)
27	1	1	1	1	1	1	1	1	1	1	36.42(0.34)

†n = 2.

The multiple regression was applied using Microsoft excel 2007 in order to deduce the factors having a significant effect on the enzymatic reaction and the best fitting mathematical model was selected. Two dimensional contour plot and three dimensional response surface plot resulting from the equations were obtained by the NCSS software.

### Incubation conditions

To define the optimal conditions for incubation and HPLC analysis, GLM (10 – 30 μMole) was incubated with HLM for 10 to 60 min across a range of microsomal enzyme concentrations (0.25 – 0.75 mg/ml). Briefly the incubation mixtures consisted of 50 mM phosphate buffer (pH 7.4), 10 mM MgCl_2_, 1 mM EDTA, 1 mM NADPH and 0.5 mg/ml of microsomal protein. In all experiments, GLM was dissolved and diluted serially in methanol and then alcohol was removed by evaporating to dryness. GLM was reconstituted in potassium phosphate buffer (50 mM, pH 7.4) .The tubes were placed into an ice bath and 5 μl of HLM was added and vortexed. Tubes (duplicate) containing the reaction mixture in phosphate buffer and NADPH solution were allowed to equilibrate separately in a shaker incubator at 150 rpm for 5 min at 37°C. The reaction was initiated by adding 20 μl of NADPH immediately to the tubes and incubation carried out for 30 min. The reaction was terminated by the addition of 100 μl ice cold acetonitrile. The tubes were centrifuged at 10,000 rpm (4°C; 10 min), and aliquots of the supernatant were directly injected into an HPLC system. Control incubations were also carried out without HLM, NADPH to confirm metabolism. Wherever necessary the volume was made up to 200 μl with buffer.

### HPLC analysis

A reported HPLC method with UV detection [22] was modified to measure GLM in microsomal incubates. The HPLC system consisted of Shimadzu LC 20 AT pump and SPD 20A UV detector, a rheodyne 7725 fixed injector loop (20 μl), Thermo scientific C18 Hypersil BDS column (4.6 x 250 mm, 5 μm) and a Phenomenex C18 guard column (4 × 3 mm). The mobile phase was composed of acetonitrile and 0.1% formic acid (55; 45 v/v). The operating temperature was ambient and flow rate was 1 ml/min. The column eluent was monitored at a wavelength of 228 nm. Under these chromatographic conditions GLM and its metabolite M1 were eluted at 3.6 and 9.3 min, respectively.

### *In vitro* metabolism of GLM using HLM

Preliminary experiments showed that the substrate depletion was linear with respect to both time over 50 min and liver microsomal protein concentration (0.3-0.65 mg/ml) at 37°C. Thus a 30 min incubation time and 0.5 mg/ml microsomal protein concentration was selected. Kinetic studies were performed by incubating eight concentrations of GLM (0-100 μMole) in duplicate with HLM.

### Determination of K_m_ and V_max_ for GLM metabolism by nonlinear and linear transformations

For the determination of the apparent Michaelis-Menten constant (K_m_) and the maximal velocity of the reaction (V_max_), plots in relation to the substrate concentration were derived using GraphPad Prism 5 software.

A number of ways of re-arranging the Michaelis-Menten equation (V = V_max_ [S]/K_m_ + [S]) have been devised to obtain linear relationships which permit more precise fitting to the experimental points, and estimation of the values of K_m_ and V_max_. Hence data for reaction velocities was also evaluated by double reciprocal plot (Lineweaver-Burk equation, 1/V = K_m_/V_max_ * 1/[S] + 1/V_max_). The intersection points were determined graphically using Microsoft Excel 2007.

### Data analysis

In the present study, the disappearance of GLM in the medium incubated at 37°C with HLM in the presence of the NADPH was determined as the percentage of the initial amount of GLM in the medium without incubation. The obtained results were expressed as the turnover rate in percentage wherever necessary. Substrate disappearance velocity was calculated as [(C_0, initial_ - C_s, t min_)/incubation time/CYP concentration], where C_0, initial_ is the substrate concentration at time 0 min and C_s, t min_ is the substrate concentration after 10, 35, 60 min incubation with 0.25, 0.5 and 0.75 mg/ml protein concentration. Metabolite formation velocity (V) was calculated as (C_s, t min_/incubation time/CYP concentration), where Cs, t min was the metabolite concentration after a 10, 35, 60 min incubation.

## Results

### Reaction linearity optimization by factorial design

Linearity of enzyme reactions in the *in vitro* human liver microsomal incubations was assessed by monitoring the effect of incubation time (from 10 to 60 min) and protein concentration (from 0.25 – 0.75 mg/ml) on metabolite formation of GLM. Using 3X3 factorial design as shown in Table 2, 27 batches were prepared varying three independent variables such as drug concentration (X1), incubation time (X2) and HLM concentration (X3). The turnover rates as response are recorded in Table 2. The results of the regression output and response of full model and reduced model are represented in Table 3. The equations for full and reduced model are given below.

**Table 3:** Response of Full Model and Reduced Model

**Turnover rate (%)**				
**Response**	**Full model**		**Reduced model**	
	**X coefficient**	**P value**	**X coefficient**	**P value**
X1	−0.903	0.093963003	-	-
X2	14.707	2.935912E-15†	14.708	1.92E-19
X3	2.891	3.72138E-05†	2.921	4.63E-06
X1^2^	−0.042	0.962269567	-	-
X2^2^	6.541	1.39313E-06†	6.541	9.21E-08
X3^2^	−3.107	0.0027451†	−3.107	0.001253
X1X2	−0.288	0.648852013	-	-
X1X3	−0.263	0.706627127	-	-
X2X3	0.468	0.46193583	-	-
X1X2X3	0.028	0.970329444	-	-
Intercept	16.522	7.07592E-11	16.49481481	5.84E-15

†significant terms at p > 0.05.

Full model

$$\text{Y}1\text{(\%)}=16.522-0.903\text{X}1+14.707\text{X}2+2.891\text{X}3-0.042\text{X}1^{2}+6.541\text{X}2^{2}-3.107\text{X}3^{2}-0.288\text{X}1\text{X}2-0.263\text{X}1\text{X}3+0.468\text{X}2\text{X}3+0.02875\text{X}1\text{X}2\text{X}3$$

Reduced model

$$\text{Y}=16.522+14.707\text{X}2+2.891\text{X}3+6.541\text{X}2^{2}-3.107\text{X}3^{2}$$

As the model was generated by taking only the significant terms from the full model, the results are deduced by interpreting the reduced model. The positive sign for coefficient of X2 and X3 in equation 1 shows that the rate of metabolism increases with increase in incubation time and HLM concentration.

The results of the Analysis of variance (ANOVA) of the second order polynomial equation are given in Table 4. F statistics of the result of ANOVA of full and reduced model confirmed omission of non-significant terms of equation 1. Since the calculated F value (0.6841) was less than the tabled F value (2.74) (α = 0.05, V1 = 6 and V2 = 16), it was concluded that the neglected terms do not significantly contribute in the prediction [23]. The goodness of fit of the model was checked by the determination coefficients (R^2^). In this case, the values of the determination coefficients (adj R^2^) were very high (>90%), which indicates a high significance of the model. All the above considerations indicate an adequacy of the regression model [24,25].

**Table 4:** Analysis of variance (ANOVA) for full and reduced models of GLM metabolism

	**DF**	**SS**	**MS**	***F*†**	**R**	**R**^**2**^	**Adj. R**^**2**^
Regression							
FM	10	4380.932	438.0932	94.570	0.9916	0.9834	0.9730
RM	4	4361.916	1090.479	257.590			
Error							
FM	16	74.119(E1)	4.632				
RM	22	93.134(E2)	4.233				

†SSE2-SSE1 = 93.134 – 74.119 = 19.015.

No. of the parameters omitted = 6.

MS of error (full model) =4.632.

*F* calculated = (SSE2 –SSE1/no. of parameters omitted)/MS of error (full model) = (19.015/6)/4.632 = 0.684189.

Tabled *F* value = 2.74 (α = 0.05, V1 = 6 and V2 = 16).

Where DF indicates degrees of freedom; SS sum of square; MS mean sum of square and *F* is Fischer’s ratio.

### Contour plot

Contour plots are a diagrammatic representation of the values of the response. They are helpful in explaining the relationship between independent and dependent variables. The reduced models were used to plot two dimension contour plot at a fixed level of 0 for X1 respectively, and the values of X2 and X3 were computed between −1 and +1 at predetermined values of the turnover rate.

**Figure 1:**
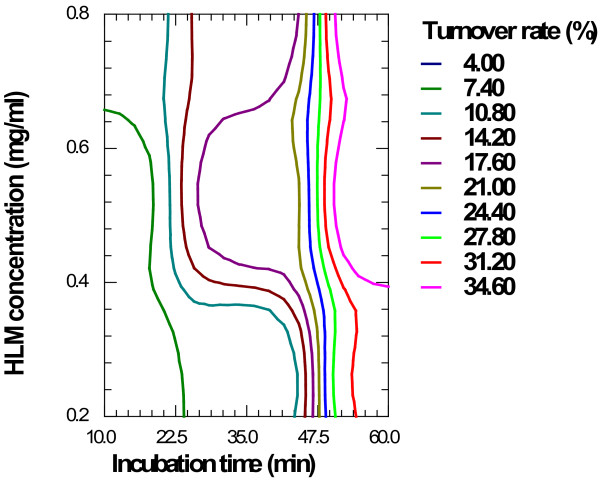
Contour plot for GLM oxidative biotransformation showing effect on turnover rate at 0 level of drug concentration (X1).

Figure 1 shows the contour plot drawn at 0 level of X_1_ (20 μMole), for a prefixed turnover rate of GLM ranging from 4.0% to 34.6%. The plot was found to be linear for approximate values of 17.60%, 21.00% and 24.40% whereas the approximate values of 10.80%, 14.20% and 17.60% showed somewhat linearly curved segments. The approximate values 7.40% and 34.60% showed inconsistent segments signifying nonlinear relationship between X_2_ and X_3_ variables. It was determined from the contour that maximum turnover of about 34.60% could be obtained with X_2_ range at 54.4 to 60 min and X_3_ at 0.4 to 0.8 mg/ml of protein concentration. As per the PhRMA and USFDA guidelines, it was observed that up to 20% metabolism of the substrate within the limits of this design could be obtained with incubation time (X_2_) from 24 to 50 min and protein concentration (X3) from 0.3 to 0.65 mg/ml. Hence for further study, 0.5 mg/ml protein and 30 min incubation time was optimized.

### Response surface plot

**Figure 2:**
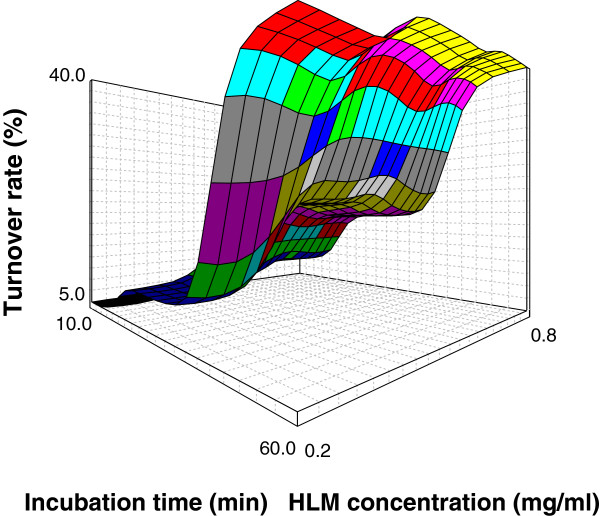
Response surface plot for GLM oxidative biotransformation showing effect on turnover rate at 0 level of X1.

Three dimensional response surface plot generated by NCSS software represented in Figure 2, depicts the turnover rate of GLM as a substrate. It shows an increase in turnover of the substrate with increase in the protein concentration and incubation time.

### Determination of K_m_ and V_max_ for GLM metabolism by nonlinear and linear transformations

**Figure 3:**
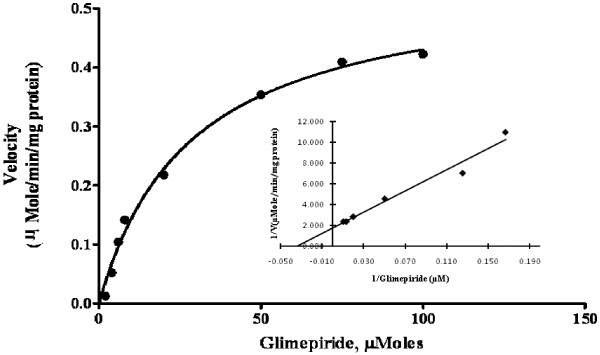
Michaelis-Menten plot for GLM oxidative biotransformation in HLM.

GLM metabolism in the presence of HLM followed Michaelis-Menten kinetics. K_m_ and V_max_ values obtained by nonlinear least squares regression method was found to be 28.9 ± 2.97 μMole and 0.559 ± 0.017 μMole/min/mg protein respectively. From Lineweaver-Burk plot the K_m_ and V_max_ values were found to be 29.411 ± 1.25 μMole and 0.571 ± 0.020 μMole/min/mg protein respectively (Figure 3). Thus the values obtained with nonlinear as well as a linear transformation of the data were found to be in close agreement with each other. Each data point represents an average of at least two parallel incubations.

## Discussion

P450 reaction phenotyping is defined as a set of experiments that aim to define which human cytochrome P450 enzyme(s) is involved in a given metabolic transformation. Such data are useful in the prediction of pharmacokinetic drug-drug interactions and interpatient variability in drug exposure. Any prolonged incubation in a closed *in vitro* system such as liver microsomes can cause formation of metabolites from the primary metabolites of a drug. Inactivation or denaturation of enzymes can become significant over time in the *in vitro* systems. Thus it is of critical importance that initial velocity conditions are defined [7].

The present study conclusively demonstrates the use of a 3X3 factorial design in the optimization of initial velocity conditions affecting turnover of GLM. The derived reduced polynomial equation, contour plot and response surface plot aid in predicting the values of selected independent variables. Contour plot (Figure 1) obtained by applying a computerized optimization process suggested a level of 30 min incubation time (X2) and 0.5 mg/ml protein (X3) as an ideal condition. At this level the turnover rate (%Y) was found to be ranging from 18.91% to 19.91%. Thus the rate of GLM disappearance was linear at the chosen concentrations of substrate using the assay conditions and detection system. However, a decrease in the level of incubation time and protein concentration below the selected level, typically yield nonlinear initial velocities of enzyme activity.

Once the optimal conditions were obtained, the substrate concentration dependence on the rate of metabolite formation was examined. The *K*_m_ and V_max_ value was determined by nonlinear regression of a plot of enzyme activity versus substrate concentration. The Michaelis constant, K_m_ accounts for the concentration of substrate at which half the active sites are filled. Thus, K_m_ provides a measure of the substrate concentration required for significant catalysis to occur. V_max_ is the rate at which substrate will be converted to product once bound to the enzyme. A substrate concentration around or below the K_m_ is ideal for determination of competitive inhibitor activity. Hence further inhibition studies are needed to confirm the performance of GLM’s oxidative biotransformation *in vitro*.

## Conclusions

This study examines the effects of the main control factors and attempts to enhance the turnover rate of GLM’s oxidative biotransformation by optimizing these factors using full factorial design. It was possible to optimize the turnover of the candidate drugs within the limits of developed assay design such that all subsequent *in vitro* incubations can be performed using the condition that ensures linearity with time and HLM concentration, and less than 20% of the initial substrate is consumed. Thus the precise information about the effects of each factor on metabolism can be used to flexibly adjust the system performance. The best estimates of K_m_ and V_max_ values were obtained with linear as well as nonlinear transformation for the enzymatic assay of GLM under initial velocity conditions.

## Abbreviations

GLM: Glimepiride; HLM: Human liver microsomes; NADPH: Nicotinamide Adenine Dinucleotide Phosphate, reduced tetra sodium salt; EDTA: Ethylene diamine tetra acetic acid; MgCl_2_: Magnesium chloride; CYP450: Cytochrome P450; PhRMA: Pharmaceutical Research and Manufacturers of America Perspective; USFDA: U.S. Food and Drug Administration; NIDDM: Non-insulin dependent diabetes mellitus; LC/UV: Liquid chromatography with ultraviolet; LC/MS: Liquid chromatography with mass spectrometry; K_m_: Michaelis-Menten constant; V_max_: Maximal velocity of the reaction; ANOVA: Analysis of variance.

## Competing interests

The authors declare that they have no competing interests.

## Authors’ contributions

DBR carried out the *in vitro* kinetic studies, participated in its design and coordination, performed the statistical analysis and drafted the manuscript. SJR has made substantial contributions for acquisition of data, its interpretation and involved in drafting the manuscript and revising it critically for important intellectual content. All authors read and approved the final manuscript.
